# Supplementary material for: Palliative Performance Scale Predicts Survival in Patients with Bone Metastasis Undergoing Radiotherapy
Source: Cancers (Basel). 2025 Dec 24;18(1):61. doi: 10.3390/cancers18010061 (PMC12784851; doi:10.3390/cancers18010061)
Supplement: Supplementary file 1 [file cancers-18-00061-s001.zip › cancers-4049193-supplementary.pdf]

# Supplementary Materials

**Table S1.** Charlson comorbidity index calculation chart [13].

| Comorbidity | Weight | Criteria |
|---|---|---|
| Myocardial infarction | 1 | History of myocardial infarction or coronary artery disease |
| Congestive heart failure | 1 | History of heart failure |
| Peripheral vascular disease | 1 | Claudication, peripheral artery disease or previous vascular surgery |
| Cerebrovascular disease | 1 | Stroke, transient ischemic attack or history of cerebral hemorrhage |
| Dementia | 1 | Clinical diagnosis of dementia |
| Chronic pulmonary disease | 1 | Chronic obstructive pulmonary disease or asthma |
| Connective tissue disease | 1 | Rheumatoid arthritis or systemic lupus erythematosus |
| Peptic ulcer disease | 1 | History of gastric or duodenal ulcer |
| Mild liver disease | 1 | Chronic liver disease without liver failure (for example, cirrhosis without ascites) |
| Diabetes without complications | 1 | Diabetes mellitus without end-organ damage |
| Diabetes with complications | 2 | Diabetes with end-organ damage, such as retinopathy or nephropathy |
| Hemiplegia or paraplegia | 2 | Paralysis due to stroke, spinal cord injury or other causes |
| Moderate or severe renal disease | 2 | Chronic kidney disease with creatinine >3 mg/dL or dialysis-dependent |
| Cancer (non-metastatic, active treatment) | 2 | Any solid tumor without metastasis, currently treated |
| Leukemia | 2 | Chronic or acute leukemia |
| Lymphoma | 2 | Non-Hodgkin's or Hodgkin's lymphoma |
| Moderate or severe liver disease | 3 | Cirrhosis with complications (ascites, encephalopathy) |
| Metastatic solid tumor | 6 | Any solid tumor with metastasis |
| HIV/AIDS | 6 | HIV infection with AIDS or opportunistic infections |

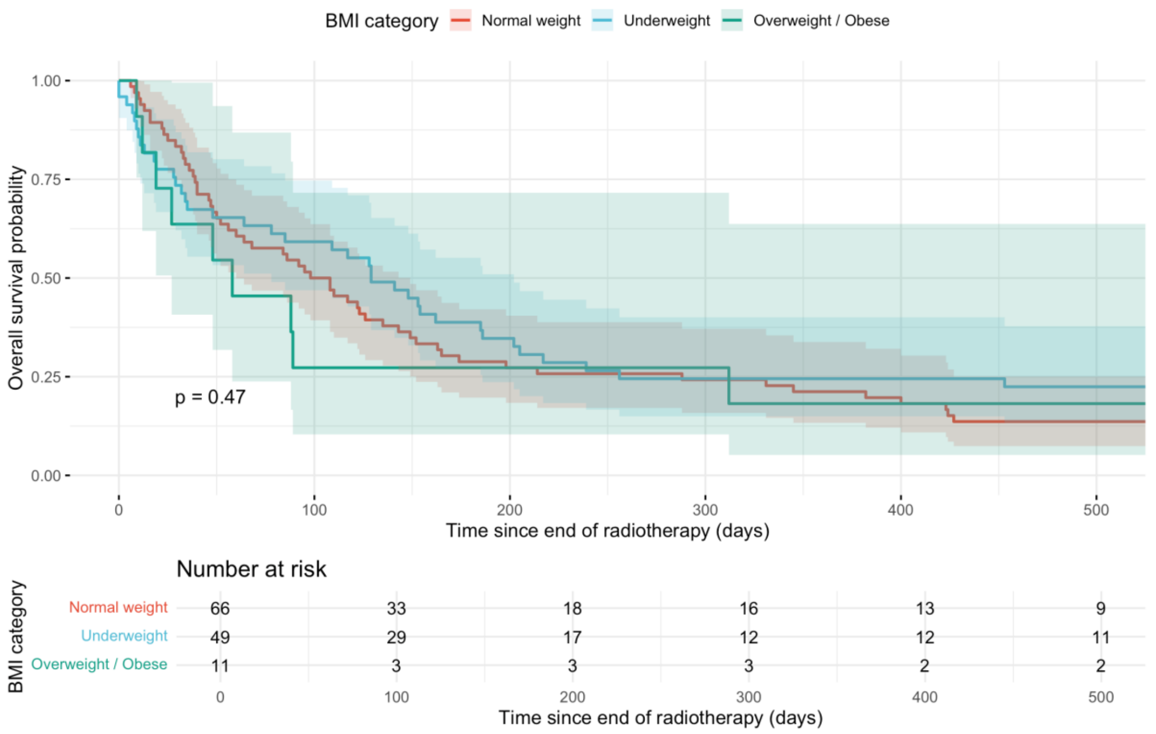


**Figure S1.** Kaplan–Meier survival curves according to BMI categories. Kaplan–Meier estimates of OS are shown stratified by BMI category (underweight, normal weight, overweight/obese) at the end of RT. No statistically significant differences in survival were observed between groups (log-rank $p > 0.05$). Abbreviations: BMI = Body Mass Index; OS = Overall Survival.

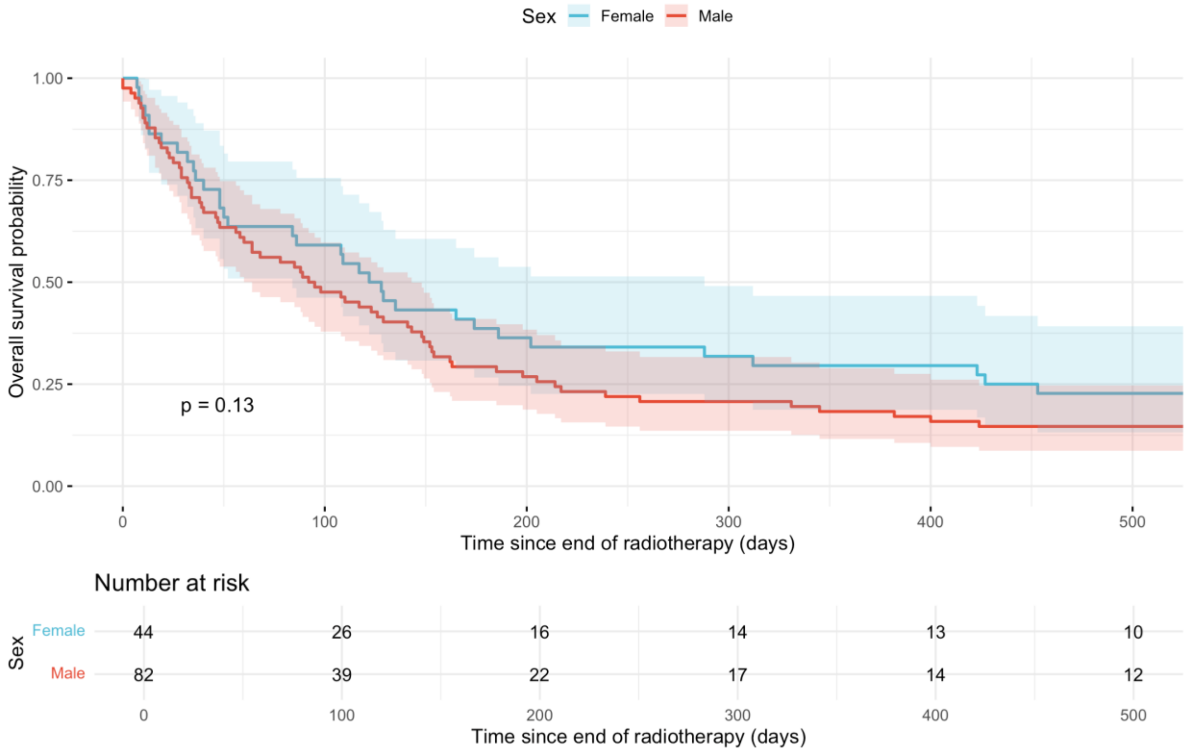


**Figure S2.** Kaplan–Meier survival curves according to sex. Kaplan–Meier estimates of overall survival (OS) are shown stratified by sex (female vs. male) at the end of radiotherapy. No statistically significant difference in survival was observed between the two groups (log-rank $p > 0.05$). Abbreviations: OS = Overall Survival.

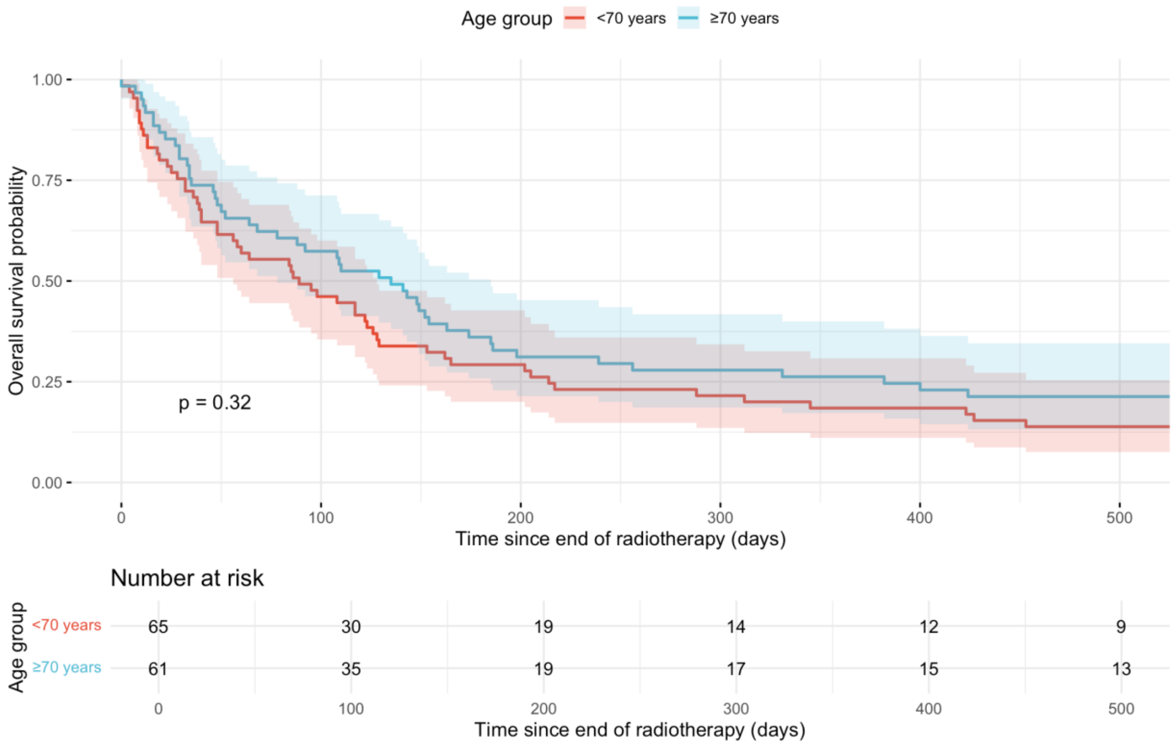


**Figure S3.** Kaplan–Meier survival curves according to age groups. Kaplan–Meier estimates of overall survival (OS) are shown stratified by age group (<70 years vs. ≥70 years) at the end of radiotherapy. No statistically significant difference in survival was observed between the two age groups (log-rank $p > 0.05$). Abbreviations: OS = Overall Survival.

## References

13. Birim, O.; Kappetein, A.P.; Bogers, A.J.J.C. Charlson comorbidity index as a predictor of long-term outcome after surgery for nonsmall cell lung cancer. *Eur. J. Cardiothorac. Surg.* **2005**, *28*, 759–762. https://doi.org/10.1016/j.ejcts.2005.06.046.
